# Human Papillomavirus Prevalence in Invasive Laryngeal Cancer in the United States

**DOI:** 10.1371/journal.pone.0115931

**Published:** 2014-12-29

**Authors:** Brenda Y. Hernandez, Marc T. Goodman, Charles F. Lynch, Wendy Cozen, Elizabeth R. Unger, Martin Steinau, Trevor Thompson, Maria Sibug Saber, Sean F. Altekruse, Christopher Lyu, Mona Saraiya, The HPV Typing of Cancer Workgroup

**Affiliations:** 1 University of Hawaii Cancer Center, University of Hawaii, Honolulu, Hawaii, United States of America; 2 Cedars-Sinai Medical Center, Los Angeles, California, United States of America; 3 Department of Epidemiology, College of Public Health, The University of Iowa, Iowa City, Iowa, United States of America; 4 Norris Comprehensive Cancer Center and Departments of Preventive Medicine and Pathology, USC Keck School of Medicine, University of Southern California, Los Angeles, California, United States of America; 5 Division of High-Consequence Pathogens and Pathology, National Center for Emerging and Zoonotic Infectious Diseases, Centers for Disease Control and Prevention, Atlanta, Georgia, United States of America; 6 Division of Cancer Prevention and Control, National Center for Chronic Disease Prevention and Health Promotion, Centers for Disease Control and Prevention, Atlanta, Georgia, United States of America; 7 Division of Cancer Control and Population Sciences, National Cancer Institute, Rockville, Maryland, United States of America; 8 Battelle Memorial Institute, Durham, North Carolina, United States of America; Georgetown University, United States of America

## Abstract

**Purpose:**

Human papillomavirus (HPV) is a major risk factor for specific cancers of the head and neck, particularly malignancies of the tonsil and base of the tongue. However, the role of HPV in the development of laryngeal cancer has not been definitively established. We conducted a population-based, cancer registry study to evaluate and characterize the genotype-specific prevalence of HPV in invasive laryngeal cancer cases diagnosed in the U.S.

**Methods:**

The presence of genotype-specific HPV DNA was evaluated using the Linear Array HPV Genotyping Test and the INNO-LiPA HPV Genotyping Assay in formalin-fixed paraffin embedded tissue from 148 invasive laryngeal cancer cases diagnosed in 1993–2004 within the catchment area of three U.S. SEER cancer registries.

**Results:**

HPV DNA was detected in 31 of 148 (21%) invasive laryngeal cancers. Thirteen different genotypes were detected. Overall, HPV 16 and HPV 33 were the most commonly detected types. HPV was detected in 33% (9/27) of women compared with 18% (22/121) of men (p = 0.08). After adjustment for age and year of diagnosis, female patients were more likely to have HPV-positive laryngeal tumors compared to males (adjusted OR 2.84, 95% CI 1.07–7.51). Viral genotype differences were also observed between the sexes. While HPV 16 and 18 constituted half of HPV-positive cases occurring in men, among women, only 1 was HPV 16 positive and none were positive for HPV 18. Overall 5-year survival did not vary by HPV status.

**Conclusions:**

HPV may be involved in the development of a subset of laryngeal cancers and its role may be more predominant in women compared to men.

## Introduction

Despite a declining incidence over the past two decades, approximately 13,000 individuals in the United States are diagnosed with invasive laryngeal cancer each year [1]. In 2010, the incidence was 5.7 and 1.1 per 100,000 in men and women, respectively. As with other malignancies of the head and neck, tobacco use and alcohol consumption are major risk factors for laryngeal cancer [2], [3]. Human papillomavirus (HPV) has been increasingly recognized as a risk factor for cancers of the head and neck [4]–[6], but the role of HPV in the development of laryngeal cancers has not been definitively established [4], [6]. HPV DNA detection in laryngeal carcinomas has ranged from 3–58% in largely single institution samples of limited size, [5], [7]–[11]. Infection of the larynx with non-carcinogenic genotypes, such as HPV 6 and 11, has been associated with laryngeal papillomatosis, a rare condition affecting both children and adults [12]. We conducted a population-based, cancer registry study to evaluate and characterize the genotype-specific prevalence of HPV in invasive laryngeal cancer cases diagnosed in the U.S.

## Materials and Methods

This evaluation was part of an initiative by the Centers for Disease Control and Prevention (CDC) to examine the distribution of HPV genotypes in anogenital and head and neck cancers diagnosed in the U.S. prior to the implementation of the prophylactic HPV vaccine [13]. This study was approved by the CDC Institutional Review Board (IRB), the IRB of the University of Hawaii Human Studies Program, the IRB of the University of Iowa, and the IRB of the University of Southern California. Individual informed consent was not obtained as study specimens were deemed to be exempt from consent related Health Insurance Portability and Accountability requirements by the NIH Office of Human Subjects Research Protections and the individual site IRBs since the de-identified, archival specimens were retrospectively collected as part of the National Cancer Institute’s Surveillance, Epidemiology, and End-Results Residual Tissue Repository program. All patient data were de-identified and anonymized prior to analysis.

Invasive laryngeal cancer was defined based on the International Classification of Diseases for Oncology Version 3 [14]. Laryngeal cancer cases were selected from patients with histologically-confirmed malignancies of any histological subtype; squamous cell carcinomas comprised the majority. All subsites of the larynx were evaluated including the supraglottis (C321), glottis (C320), subglottis (C322), and overlapping and unspecified lesions (C328 and C329). Cases were diagnosed in 1993–2004 within the catchment area of three population-based cancer registries. The Hawaii Tumor Registry, the Iowa Cancer Registry, and Los Angeles County Cancer Surveillance Program are part of the National Cancer Institute’s (NCI) Surveillance, Epidemiology, and End-Results (SEER) program and operate affiliated Residual Tissue Repositories (RTR) [15]. The RTRs consist of a collection of formalin-fixed, paraffin-embedded (FFPE) malignant tumor (herein referred to only as “tumor”) tissue specimens from cancer patients diagnosed within the catchment area of the three registries. Through linkage with registry data, tissue specimens were annotated with de-identified, high-quality demographic, clinical, treatment, and pathologic data. Stage was based on the SEER staging classification system defining the extent of disease involvement as localized, regional spread, and distant metastases [16]. Vital status and length of survival were available for a subset of cases.

FFPE tissue specimens were prepared at each site following a uniform protocol to avoid cross-contamination. A representative block from each cancer case was selected and sectioned using a new disposable blade for each case. The first and last sections were stained with hematoxylin and eosin (H&E) and intervening sections (10 microns) were transferred into 2 ml conical tubes (Simport, Beloeil, Canada). H&E sections were reviewed by a study pathologist (E.R.U.) at the CDC to confirm the presence of malignancy.

HPV genotyping was conducted at the CDC laboratories. One suitable tissue sample from each case was processed using high temperature assisted tissue lysis [17] and automated DNA purification with a Chemagic MSM1 (PerkinElmer, Waltham, MA, USA). The resulting 100 µL DNA eluate was tested immediately or stored at −20°C until testing. A blank sample without tissue was included in every sample batch to monitor potential cross contamination.

HPV testing was performed as previously described [13]. Briefly, all samples were tested using the Linear Array HPV Genotyping Test (LA, Roche Diagnostics, Indianapolis, IN), a PCR-based assay targeting a 450-bp consensus region of the L1 HPV genome and detecting 37 HPV genotypes (6, 11, 16, 18, 26, 31, 33, 35, 39, 40, 42, 45, 51, 52, 53, 54, 55, 56, 58, 59, 61, 62, 64, 66, 67, 68, 69, 70, 71, 72, 73, 81, 82, 83, 84, 89, IS39). Amplification of the human β-globin gene was included as an internal control. Samples testing negative for β-globin and HPV in the Roche assay were subsequently tested using the INNO-LiPA HPV Genotyping Assay (LiPA, Innogenetics, Gent, Belgium), a PCR-based assay with an internal control and primers targeting a 65 bp region of the HPV L1 gene. LiPA detects 26 of the same types covered in the Roche assay in addition to 3 additional types (HPV 43, 44, 74). This second assay was included in order to account for the fixation-related degradation of DNA extracted from FFPE tissue. Specimens testing negative for HPV and the internal control in both two assays were considered inadequate for evaluation.

Statistical analyses were conducted using SAS version 9.2. Overall HPV prevalence was based on the detection of one or more HPV genotypes in tumor tissue. Multiple genotypes detected in a case were not counted more than once in overall prevalence estimates. HPV 16, 18, 31, 33, 35, 39, 45, 51, 52, 56, 58, 59, 66, and 68 were considered carcinogenic, or high-risk genotypes [18], [19]. All other genotypes were considered to be non-carcinogenic, or of low or undetermined risk. Comparisons between HPV-positive and HPV-negative tumors were made using the Chi-squared statistic for discrete variables. Unconditional logistic regression was used to estimate odds ratios (OR) and 95% confidence intervals (CI). Variables with significance level <0.10 in univariate analysis were included in multivariate models; backward elimination was used to select the final model. Survival analyses excluded 22 cases for which vital status and length of survival were not available. Survival was based on the time period from date of cancer diagnosis to date of death or date of last follow-up. Overall five-year survival by HPV status was evaluated using the Kaplan-Meier method and the Log rank-test. Cox proportional hazards regression was used to examine the relationship of HPV status and covariates with overall 5-year survival. Age, which was imputed to the mid-point of 5-year age groups for 48 of the 148 cases for which single age was not available, was included as a continuous covariate in all models. All tests were two-sided and a *P* value<0.05 was considered to be statistically significant.

## Results

Tumor tissue specimens from 179 cases of invasive laryngeal cancer were initially available for the study. Of these cases, 1 was excluded due to poor quality of the tissue specimen. Of the 178 cases that were analyzed for HPV DNA, 30 specimens testing negative for both HPV and the internal control were excluded. The remaining 148 cases yielding valid HPV results were included in the present analyses. The 30 cases with inadequate HPV results were comparable to the 148 cases in the final study sample with respect to demographic, clinical, and pathologic characteristics (data not shown) indicating that no bias was introduced with the exclusion of these cases.

Men comprised 82% and women 18% of laryngeal cancer patients (**Table 1**). Seventy-one percent of cases were age 60 years and older at diagnosis. Patients were predominantly white (72%), followed by Asians (18%), Pacific Islanders (7%), and blacks (3%). The majority of cases (62%) were diagnosed in 1993–1998. Early stage (localized) tumors constituted 67% of cases. Sixty-six percent of cases were of moderately well-differentiated grade. The primary subsite location of tumors was the glottis, which constituted 62% of cases. Squamous cell carcinoma (SCC) of unspecified histological subtype comprised 83% of tumors.

**Table 1:** Characteristics and HPV status of invasive laryngeal cancer cases (n = 148).

	No.	%
*Sex*		
Male	121	81.8
Female	27	18.2
*Age at diagnosis (years)*		
<60	43	29.1
≥60	105	70.9
*Race/ethnicity*		
White	106	71.6
Asian	26	17.6
Pacific Islander	11	7.4
Black	5	3.4
*Year of diagnosis*		
1993–1998	91	61.5
1999–2004	57	38.5
*Stage* 1 ^,^ 2		
Localized	93	66.9
Regional involvement	29	20.9
Metastatic	17	12.2
*Grade* 3		
Well-differentiated	17	12.6
Moderately differentiated	89	65.9
Poorly differentiated/undifferentiated	29	21.5
*Subsite*		
Glottis	92	62.2
Supraglottis	45	30.4
Subglottis and other subsites^4^	11	7.4
*Histology*		
Squamous cell carcinoma (SCC), unspecified subtype	123	83.1
Keratinizing SCC	15	10.1
Large cell non-keratinizing SCC	4	2.7
Other^5^	6	4.0
*HPV*		
negative	117	79.1
positive	31	20.9
*carcinogenic HPV*		
HPV 16	9	6.1
HPV 18	3	2.0
HPV 31	1	0.7
HPV 33	9	6.1
HPV 35	2	1.4
HPV 39	1	0.7
HPV 51	3	2.0
HPV 66	2	1.4
*non-carcinogenic HPV*		
HPV 6	2	1.4
HPV 11	1	0.7
HPV 54	1	0.7
HPV 70	1	0.7
HPV 89	1	0.7
HPV X^6^	2	1.4

1 Based on the SEER staging classification system defining the extent of disease involvement as localized, regional spread, and distant metastases.

2 Excludes 9 cases for which data on stage are missing.

3 Exclude 13 cases for which data on grade are missing.

4 Overlapping and unspecified lesions of the larynx.

5 Includes papillary SCC, spindle cell SCC, basaloid SCC, small cell neuroendocrine carcinoma.

6 HPV amplicon not hybridizing to any of the type-specific probes in the assay.

HPV DNA was detected in 21% of laryngeal cancer cases (**Table 1**). Thirteen different genotypes were detected. Twenty-five of the 31 HPV-positive tumors contained a single genotype. Carcinogenic HPV types (alone or in combination with non-carcinogenic types) were detected in 26 of the 31 HPV-positive tumors. Carcinogenic HPV 16 and HPV 33 were the most commonly detected genotypes. These two genotypes were each detected in 9 tumor specimens including 6 cases in which they were the sole genotype. Other carcinogenic genotypes included HPV 18, HPV 31, HPV 35, HPV 39, HPV 51, and HPV 66. Non-carcinogenic HPV types included HPV 6, HPV 11, HPV 54, HPV 70, and HPV 89. Two cases were HPV X, the designation used for an HPV amplicon that did not hybridize to any of the type-specific probes in the assays.

The relationship of HPV status with case characteristics was evaluated (**Table 2**). In crude analyses, nonsignificant differences (0.05≤*P*≤0.10) were observed by sex, year of diagnosis, and stage. HPV was detected in 33% of tumors in women compared with 18% of tumors in men (*P* = 0.08). Of patients diagnosed in 1999–2004, 28% were HPV-positive compared with 17% diagnosed in 1993–1998 (*P* = 0.09). HPV was detected in 30% of advanced staged cancers and 17% of early stage cancers and (*P* = 0.07). Comparisons of HPV status by histological classification were not possible due to limited subtype numbers.

**Table 2:** Relationship of HPV and laryngeal cancer case characteristics.

	HPVDNA+	(n = 31)	HPVDNA-	(n = 117)					
	No.	%	No.	%	P value	UnadjustedOdds ratio	95% CI	Adjusted^1^Odds ratio	95% CI
*Sex*									
Male	22	71.0	99	84.6	0.08	1.00 (ref)			
Female	9	29.0	18	15.4		2.25	0.89–5.67	2.84	1.07–7.51
*Age at diagnosis (years)*									
<60	20	64.5	85	72.6	0.37	1.00 (ref)			
≥60	11	35.5	32	27.4		0.68	0.30–1.59	0.97	0.93–1.01
*Race/ethnicity*									
White	22	71.0	84	71.8	0.93	1.00 (ref)			
Non-White	9	29.0	33	28.2		1.04	0.44–2.55	0.66	0.23–1.89
*Year of diagnosis*									
1993–1998	15	48.4	76	65.0	0.09	1.00 (ref)			
1999–2004	16	51.6	41	35.0		1.98	0.89–4.40	2.15	0.93–4.96
*Stage* 2 ^,^ 3									
Localized	16	53.3	77	70.6	0.07	1.00 (ref)			
Regionalinvolvement/metastatic	14	46.7	32	29.4		2.11	0.92–4.82	1.55	0.64–3.73
*Grade* 4									
Well-/moderately differentiated	23	76.7	83	79.0	0.78	1.00 (ref)			
Poorlydifferentiated/Undifferentiated	7	23.3	22	21.0		1.15	0.44–3.02	1.05	0.38–2.87
*Subsite*									
Glottis	20	64.5	72	61.5	0.76	1.00 (ref)			
Supraglottis,subglottis, other^5^	11	35.5	45	38.5		0.88	0.39–2.01	0.65	0.26–1.59

1 Adjusted for covariates in the final multivariate model (sex, age, year of diagnosis).

2 Based on the SEER staging classification system defining the extent of disease involvement as localized, regional spread, and distant metastases.

3 Excludes 9 cases for which data on stage are missing.

4 Exclude 13 cases for which data on grade are missing.

5 Overlapping and unspecified lesions of the larynx.

Sex (male, female), age (continuous), stage (localized, regional/metastatic), and year of diagnosis (1993–1998, 1999–2004) were included in the initial multivariate model. In the final multivariate model adjusted for age and year of diagnosis, sex remained a significant predictor of HPV positivity: Female patients were more likely to have HPV-positive laryngeal tumors compared to males (adjusted OR 2.84, 95% CI 1.07–7.51).

There was no difference in the proportion of specimens requiring testing with the LiPA assay for cases diagnosed before 1999 (26%) and those diagnosed in 1999–2004 (40%) (*P* = 0.08) indicating that the observed differences in HPV detection by time period were not attributed to greater degradation of older specimens. Registry site was not associated with HPV detection in univariate or multivariate models (individual registry data not shown in the interest of confidentiality).

Given the significant association of female sex and HPV positivity, characteristics of cases were examined by sex. Notably, more female laryngeal cancer patients were white (24/27, 89%) than male patients (82/121, 68%) (*P* = 0.03). The majority of tumors in women were located in the supraglottis (16/27, 59%) while male tumors were predominantly located in the glottis (83/121, 69%) (*P* = 0.001).

**Figure 1:**
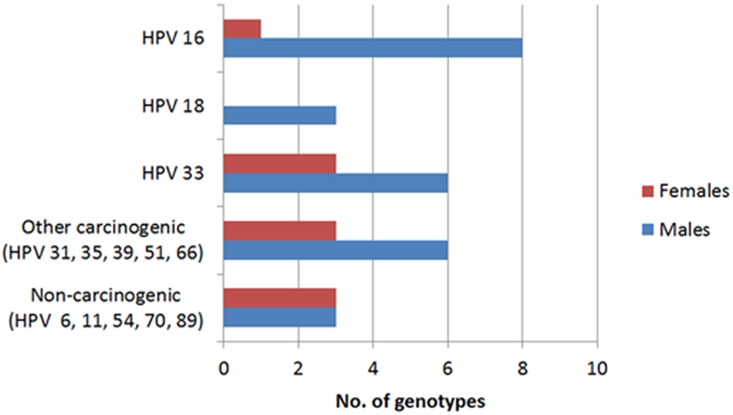
Distribution of HPV genotypes in HPV positive tumors (male n = 22; female n = 9).

HPV genotype distribution varied widely in men and women (**Fig. 1**). In men, HPV 16 was the most frequently detected type followed by HPV 33 and HPV 18. In contrast, HPV 33 was the most common type in women followed by other carcinogenic and non-carcinogenic types. Only 1 female case was positive for HPV 16 and none were positive for HPV 18.

**Figure 2:**
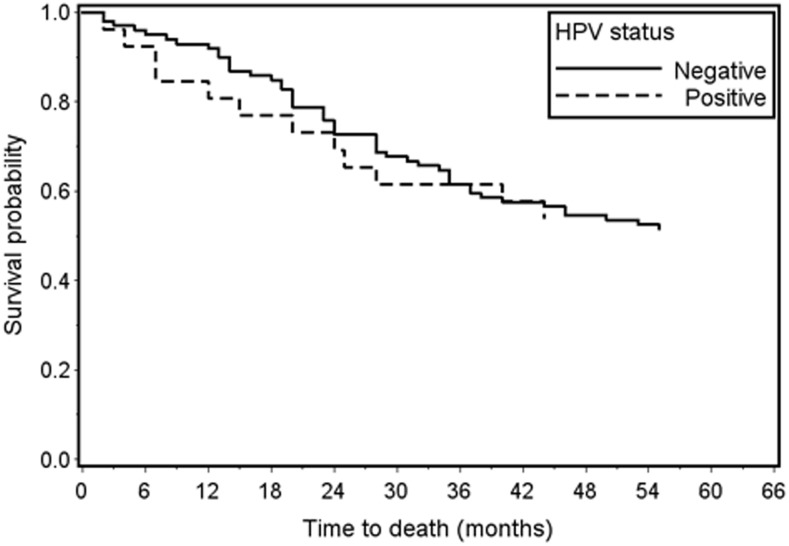
Overall 5-year survival in laryngeal cancer cases by HPV status (Log-rank *P*-value = 0.88; HPV+ n = 26; HPV− n = 100).

Overall 5-year survival was comparable by HPV status: 54% for HPV-positive cases and 52% for HPV-negative cases (log-rank *P* value = 0.88) (**Fig. 2**). Glottic tumors (vs. non-glottic) and early stage (vs. advanced stage) were each positively associated with 5-year survival in univariate analyses. Adjusting for these covariates in multivariate models, HPV status was not associated with 5-year survival (adjusted hazards ratio 1.28, 95% CI 0.66–2.51); tumor subsite and stage were no longer significant.

## Discussion

Our study results indicate that HPV may be involved in the development of a subset of laryngeal cancers. To our knowledge, the present study is one of the largest U.S.-based samples of invasive laryngeal carcinomas to be evaluated for HPV [5]. In a review of 35 studies of laryngeal cancers, Kreimer *et al*. reported an average detection rate of 24% [5], which is very close to our observed estimate of 21%.

Compared to men, women were more likely to have HPV-positive laryngeal cancers after controlling for potential confounding factors. Other differences by sex were observed. Female laryngeal cancer patients were comprised of greater proportion of Whites than male patients. Tumors in women were more likely to be located in the supraglottis while those in men were predominantly in the glottis. This sex-specific difference in the location of laryngeal tumors has been previously recognized [20]. Nonetheless, HPV status did not vary by race or subsite indicating that these factors did not account for differences in HPV status between the sexes. Overall, HPV 16 and HPV 33 were detected with equal frequency and were the most common types detected in laryngeal cancers. Carcinogenic types other than HPV 16 and 18, however, were more frequent in tumors occurring in women.

Our results are supported to some extent by a study of 69 cases of *in* *situ* and early stage laryngeal carcinomas in a U.S. medical facility in which HPV was detected in 33% of tumors of women compared with 10% of tumors of men [21]. In contrast, a study of 79 laryngeal cancer cases in another U.S. medical facility reported a higher proportion of HPV in laryngeal tumors of men than women [22].

Our assessment of HPV exposure was limited to viral detection in tumor tissue. Therefore, we are unable to establish whether HPV infection preceded the development of laryngeal cancer, which would be critical to the determination of causality. Our findings would be bolstered by evidence of HPV in precancerous laryngeal tissue or serologic evidence of infection prior to cancer diagnoses.

Another limitation of the present analysis is the lack of information on tobacco and alcohol use as well as sexual history. Accordingly, our inability to account for differences in tobacco and alcohol use in our study population limits our ability to draw conclusions regarding potential sex differences in HPV-induced laryngeal carcinogenesis. Tobacco use and alcohol consumption are among the most important risk factors for head and neck cancers [3], [23]. HPV-negative head and neck cancers are more closely associated with tobacco and alcohol use while HPV-positive tumors are related to sexual exposures [2], [24]. It is possible that the observed differences between the sexes are explained by differences in alcohol and tobacco use. Nonetheless, there is little evidence for gender differences in the association of smoking and alcohol consumption with risk of laryngeal cancer [25]. Moreover, unlike oropharyngeal cancers, including the tonsil and base of the tongue, laryngeal cancers have not been tied to sexual history [26].

Historically, the incidence of laryngeal cancer in men far exceeds that in women [27]. The incidence of laryngeal cancers has been decreasing over the past several decades in the U.S. and in other parts of the world, largely due to the decreases in the prevalence of smoking [27]–[31]. Nonetheless, the declining rates have predominantly occurred in males, while the incidence in women has increased over time [27]–[31]. Although speculative, it is possible that HPV may be influencing the increasing rates in females. Chaturvedi *et al* [32] reported an increase in HPV-positive oropharyngeal cancers in the U.S. over time but no increase in HPV-negative tumors. This study included tumor samples from the same three SEER registries included in the present analysis. We also found some evidence that HPV-positive laryngeal tumors were more common in more recently diagnosed cases as over half of HPV-positive laryngeal cancers were diagnosed after 1998, although the differences were non-significant. While differences in tissue processing and preservation cannot be entirely excluded as an explanation for the decreased detection of HPV in older tissues, we found no difference in preservation based on proportion of tissues that required use of the short-fragment LiPA for HPV testing.

We observed no association of HPV status with overall 5-year survival in laryngeal cancer. For other cancers of the head and neck, HPV tumor positivity favorably influences outcome, including overall survival, disease-free survival, and recurrence [32]–[48]. The survival advantage of HPV has primarily been shown for oropharyngeal cancers, particularly tonsillar tumors [33], [35], [37], [39], [45]–[47] and, to a lesser extent, for oral cancers [37], [41], [44]. The reasons for better survival associated with HPV tumor positivity are not entirely clear but may be due to patient differences or treatment-related effects. HPV-positive oropharyngeal cancer patients are more likely to be younger individuals without a history of tobacco and alcohol use [49]. There is also evidence that HPV-positive oropharyngeal tumors are more susceptible to chemotherapy agents [33], [46] and HPV-positive patients experience enhanced immune response following radiotherapy [50], Our findings of no survival advantage in HPV-positive laryngeal cancers are consistent with a recent study [51] and suggest that HPV may not have the same prognostic significance in laryngeal cancers as with oropharyngeal malignancies. Nevertheless, our ability to comprehensively discern the relationship of HPV and survival was limited by the lack of information on treatment history as well as tobacco and alcohol use.

In summary, our study results provide evidence that a subset of invasive laryngeal cancers may be caused by HPV. Moreover, our results suggest that HPV may be a more important cause of laryngeal tumors in women. Nonetheless, our conclusions should be considered preliminary as our results do not prove that HPV plays a causal role in laryngeal carcinogenesis. Detection of HPV DNA in a cross-sectional analysis is insufficient to determine causality. Our results ideally would be confirmed in longitudinal studies following cohorts with asymptomatic HPV detection and with precursor lesions progressing to cancer, similar to those performed establishing the link between cervical cancer and HPV. Additional studies examining tobacco, alcohol, and sexual history, as well as molecular markers such as HPV DNA copy number, E6/E7 mRNA, and p16 would also be important in order to support the etiologic role of HPV in laryngeal cancer.
